# The application of the BOPPPS model in the ward rounds of nurses’ standardized training in Southwest China: a mixed methods study

**DOI:** 10.3389/fmed.2024.1276652

**Published:** 2024-06-27

**Authors:** Ying Shen

**Affiliations:** Nursing Department, The First Affiliated Hospital of Chongqing Medical University, Chongqing, China

**Keywords:** BOPPPS model, clinical practice, education assessment, medical education, nurse standardized training

## Abstract

**Background:**

Teaching ward rounds are the main teaching method used to develop clinical skills in standardized nursing training. However, the existing methods lack of cultivation of comprehensive ability and humanistic care for nurses, cannot meet the requirements of standardized training for nurses. BOPPPS (bridge-in, objective, pre-assessment, participatory Learning, post-assessment, and summary) is a student-centered teaching model that has been proven to enhance classroom teaching effectiveness. Therefore, the BOPPPS model was applied and its effectiveness in standardized nursing training was evaluated.

**Methods:**

In total, 260 nursing students were randomly allocated to two groups: the experimental group used the BOPPPS model and the control group used the traditional teaching model. This study used a mixed quantitative and qualitative research method to evaluate the effectiveness of the BOPPPS model.

**Results:**

The quantitative results were as follows: no significant difference in baseline scores was observed between the two groups before training. After training, the theory and practical scores in the experimental group were significantly higher than that of the control group. Similarly, students in the experimental group presented higher comprehensive ability scores than their counterparts. The students in the experimental group also exhibited higher satisfaction compared to the control group, while there was no difference in teacher satisfaction scores between the two groups (*p* = 0.323). Qualitative data showed that the vast majority of nurses and teachers agreed on the value of BOPPPS training.

**Conclusion:**

Compared to traditional teaching methods, the BOPPPS model was more effective in standardized nursing training. We recommend applying the BOPPPS model to nursing training.

## 1 Introduction

Newly graduated registered nurses (NGRNs) in China undergo 2 years of standardized training to help nurses better adapt to clinical roles and provide comprehensive and high-quality holistic care for patients. The regulations involve 6 months of training in the surgery (cardiothoracic surgery, general surgery, orthopedics, etc.) to enhance their clinical skills and expertise (1–3). At present, there is no unified teaching model standard in China, and traditional teaching models are commonly used. However, the traditional teaching model is teacher-centered, emphasizes knowledge teaching, and lacks a personalized approach to the characteristics of the nursing students, which may inhibit their learning initiative and enthusiasm (4, 5). In addition, the traditional education model does not consider the differences in the students’ knowledge and individual learning abilities, ignoring individual differences among students and failing to facilitate their personal growth (6–8). A new and valid teaching model is essential for the training of GNRNs.

The BOPPPS teaching strategy originated from the Canadian Teacher Skills Training Workshop (Instructional Skill Workshop, ISW), which is a teaching model based on cognitive theory (9). The BOPPPS model is divided into six distinct steps, with BOPPPS stands for bridge-in, objective, pre-assessment, participatory learning, post-assessment, and summary. Bridge-in is performed by arousing student curiosity, stimulating their interest in learning, appropriately introducing learning content, and establishing a good learning motivation at the beginning of learning. The objective is determined through learning, highlighting the cognitive, emotional, or skill ability level that the students should be able to achieve. Pre-assessment is an evaluation of what students have learned, which guides the follow-up classroom teaching design and classroom activities. Participatory learning emphasizes the deep participation of students in the learning process. Post-assessment determines whether the students have grasped the content after the class, and evaluates whether the expected teaching objectives have been achieved. Summary mainly allows teachers and students to summarize and reflect on the teaching. Teachers can reflect on the experience and efficiency of the teaching, while students reflect on their challenges and gains in the learning process. The BOPPPS model provides a clear teaching process and induces students to take learning initiatives (10). The BOPPPS teaching model has been widely applied in medical education. Li et al. (11) found that combining BOPPPS and team-based learning can improve the self-learning and critical thinking abilities of nursing undergraduates. Xu et al. (12) explored the application effect of the mixed BOPPPS teaching model in gynecological clinical internships and demonstrated that the BOPPPS teaching model could stimulate students’ learning interest and initiative, enhance their clinical practice ability, and improve their satisfaction. The BOPPPS model has also been applied to practical teaching in other disciplines, including physiology education (13), electrocardiogram teaching (14), ophthalmology education (7) and health services management (15).

Nevertheless, no studies have assessed the application of the BOPPPS model in the training of NGRNs, whether in China or other countries. Hence, this research sought to evaluate the effectiveness of the BOPPPS model in training NGRNs in Southwest China.

## 2 Methods

This study employed a mixed-methods approach, using both quantitative experimental and descriptive qualitative methodologies in a complementary design. The qualitative findings were used to provide further insight and explanation for the quantitative results. Based on the research purpose, the observation indicators revolved around the teaching/learning process, results, and personal feelings.

### 2.1 Inclusion and exclusion criteria

Participants included new nurses with standardized training at the First Affiliated Hospital of Chongqing Medical University from July 2017 to June 2022, and these nurses graduated from universities or vocational colleges in Chongqing, Sichuan, Guizhou, and Yunnan. We excluded nursing students following online courses during the epidemic period. According to the hospital training plan, the students were divided into groups of 5–8 students and were assigned to department rotations of 3 months each. The decision was made by a third party who did not participate in the study by tossing a coin, with the odd numbers as the experimental group and the even numbers as the control group. Both groups met the “Chongqing Nursing Standardized Training Standard” (16).

According to the training program, the objectives of the teaching ward rounds were pulmonary nodule resection patients 2 days after the procedure. The teachers were the same between both groups and were responsible for preparing the syllabus and the teaching materials. In addition, they checked the case reports, ward round teaching aids, communicated with the patient in advance, and obtained consent.

### 2.2 Teaching process design

#### 2.2.1 Experimental group

**Figure 1:**
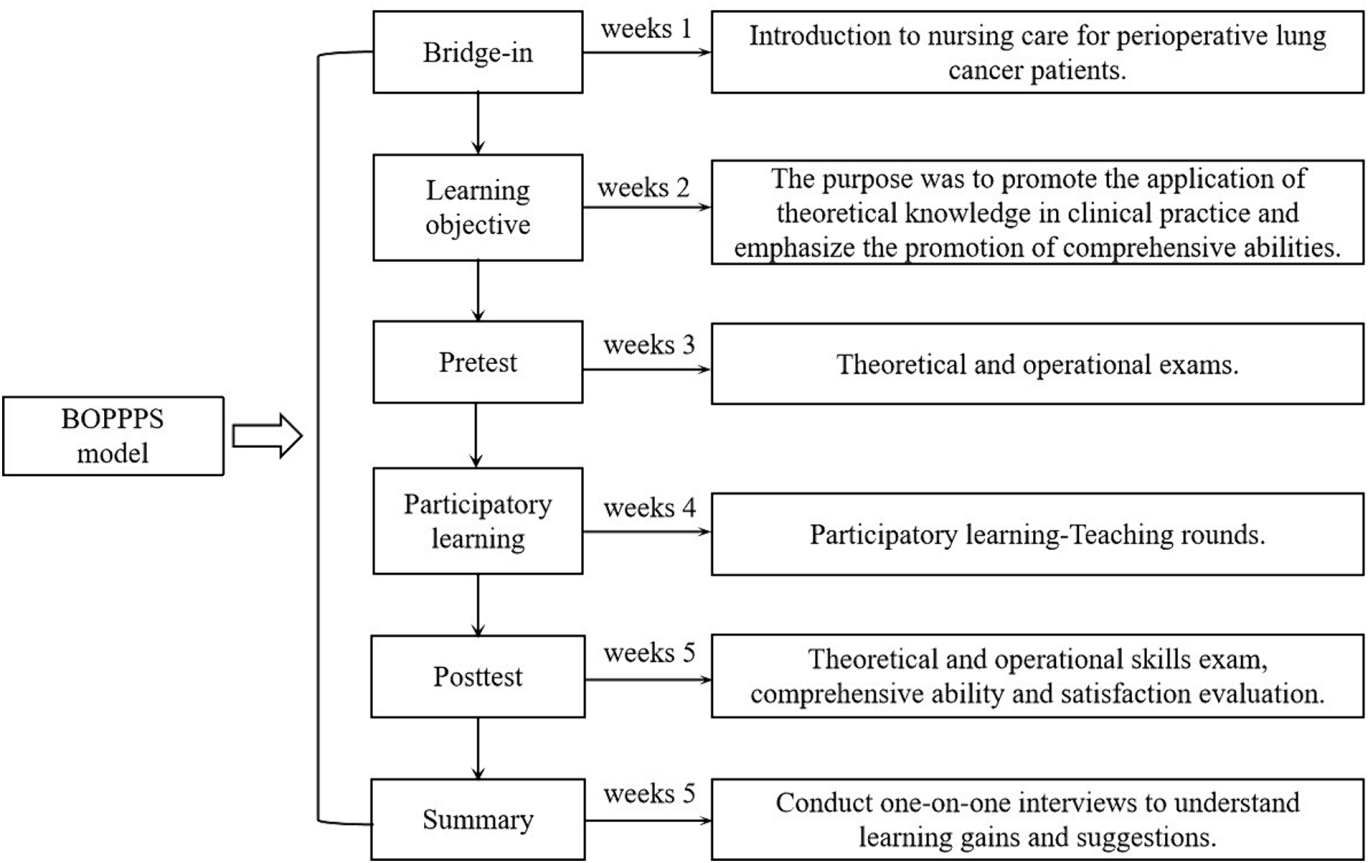
Flowchart design for BOPPPS Model.

The experimental group applied the BOPPPS teaching model in ward rounds. The teaching model was based on group cooperation, with nursing students taking “lecturer” roles (Table 1) and emphasizing their participation in the learning process (Participatory learning). The teacher assisted the “lecturer” by asking questions and initiating discussions to prompt students to think actively. The specific process was as follows (Figure 1):

**Table 1:** Role assignment and division of labor of nursing students in the experimental group.

Role	Content
Headman	Cooperate with the main ward round teachers to coordinate and implement the relevant work during the teaching rounds.
Responsible nursing students	Under the guidance of the teacher, record the required information for the medical history report, including basic patient information, past history, current medical history, test results, related treatment, and current nursing problems and measures.
Physical examination	Conduct physical examinations based on the specific situation of the patient, highlighting key points.
Recorder	Record the process of teaching activities, and complete the record and sorting under the guidance of teachers.
New progress report	Present the progress in disease treatment.

##### 2.2.1.1 Week 1

Introductory cases (Bridge-in) were presented to stimulate students’ curiosity and interest in learning, and guide nursing students to observe and learn purposefully before teaching rounds.

##### 2.2.1.2 Week 2

The learning Objectives (Objective) were displayed on the blackboard, and the teacher elaborated on the specific requirements. This exercise promoted the application of theoretical knowledge in clinical practice and emphasized the development of comprehensive abilities. It is beneficial for nursing students to complete the preparation of theoretical knowledge and operational skills according to their own situation before ward rounds.

##### 2.2.1.3 Week 3

Pretest (Pre-assessment) was performed by arranging homework before the teaching round. The students were required to review Chapter 21 Section 1 Lung Cancer of the 9th edition of “Surgery, Nursing” within 1 week, including knowledge of lung anatomy, common surgical methods, nursing diagnosis and measures, and auscultation methods of lung respiratory sounds.

A theoretical test was conducted based on the preschool homework to evaluate the preparation of the nursing students, which allowed the identification of knowledge gaps for a more thorough understanding of the subject. The breathing sound auscultation training was guided one-on-one by a separate teacher, and the teacher of the operation group was responsible for the evaluation and correction.

##### 2.2.1.4 Week 4

Participatory ward rounds (Participatory learning, 2 credit hours). Task assignment was given 2 days in advance (Table 2).

**Table 2:** Process arrangement of teaching round in the experimental group.

Place	Role	Content	Method	Time
Study room	Teaching ward rounds teacher	Knowledge review and investigation.	Asked questions and supplemented the content of preschool self-study.	5 min
Study room	Responsible nursing students	Report the basic patient information, current medical history, past history, and current treatment.	The teacher guided the nursing students to self-evaluate and assess the completeness and accuracy of the reported information, and finally supplemented and summarized the case.	15 min
Bedside	Physical examination	Physical examination, including inspection, palpation, percussion, auscultation, communication skills, and health education.	The nursing students mainly assisted in the physical examination. After the physical examination, the students were prompted to self-evaluate in the form of questions.	20 min
Study room	Responsible nursing students	Report on the patient’s current nursing problems, related factors, and nursing measures.	The students discussed the report in the form of questions, with a final summary by the teacher.	25 min
Study room	New progress report students	Present the literature review results.	Guided nursing students to discuss, combined with supplementary clinical instructions from the teachers.	10 min
Study room	Teacher	Final comments	Final comments on this teaching round.	5 min

##### 2.2.1.5 Week 5

Posttest (Post-assessment, Week 5):

###### 2.2.1.5.1 The theory and practical skills exam

The theoretical knowledge was assessed by the department’s examination paper, and the practical skills (respiratory sound auscultation) were evaluated by the teachers according to the evaluation standards of the School of Nursing, Chongqing Medical University.

###### 2.2.1.5.2 Comprehensive ability evaluation

A comprehensive competency evaluation scale was developed based on the standardized nurse training and evaluation standards of the First Affiliated Hospital of Chongqing Medical University. The scale comprised 11 aspects and covered learning initiative, participation in ward rounds, knowledge mastery of ward rounds, critical thinking ability, improvement of practical ability, etc. Evaluation by the teaching teacher based on the performance of students in teaching rounds. A 5-point Likert 5 scale was used, with a total score of 11 ~ 55 points; a higher score indicated a higher comprehensive clinical competence.

###### 2.2.1.5.3 Satisfaction evaluation

A self-designed satisfaction evaluation form was used to investigate the satisfaction of nursing students and clinical teachers. The survey content included curriculum design, student/teacher performance, work/learning pressure, teaching/learning outcomes, and overall evaluation. A 5-point Likert 5 scale was used, with a total score of 20 ~ 100 points; a higher score indicated a higher satisfaction level.

##### 2.2.1.6 Summary (week 5)

Nursing students completed the reflection diary and summarized what they learned during the teaching rounds. The reflection diaries were reviewed by the teacher, the teaching activity was recorded, and the experience and shortcomings of this teaching session were examined. Finally, the design of the next teaching round was planned.

#### 2.2.2 Control group

##### 2.2.2.1 Preliminary preparation (first week)

Teachers collected information, consulted literature related to the ward rounds, prepared ward round teaching materials, and organized the teaching rounds. Nursing students were informed to review the content of the ward rounds.

##### 2.2.2.2 Teaching rounds (week 2, 2 credit hours)

The teacher briefly explained the relevant knowledge points (5 min). The responsible nursing student reported the medical history (15 min) and performed the bedside physical examination (20 min). Subsequently, the teacher provided comments and supplementary information (10 min). Finally, the teacher guided the nursing students to discuss nursing diagnosis, influencing factors, and nursing measures in the study room (20 min), and summarized the knowledge points based on the problems reflected during the ward round (10 min).

The pre-assessment, post-test, and summary steps were the same as the experimental group.

### 2.3 Interviews in the qualitative phase

Data collection was conducted through semi-structured interviews. Qualitative results were used to explain quantitative research findings and further improve the teaching model of BOPPPS. The interview was conducted in a face-to-face manner, with the teacher conducting individual interviews and the students conducting focus group interviews. To mitigate potential bias in the findings, the interview was administered by the hospital’s educational assessment team, comprising two interviewers tasked with conducting interviews with nurses and educators. Before the interview, the interviewers underwent training to familiarize themselves with the objectives and protocols of the study. All interviews was transcribed by the two individuals within 24 h, and any ambiguities was resolved by a third party. Additionally, all participants were informed as to the aims of the study and volunteered to participate before the interview. The interview lasted for 22–30 min and was conducted in quiet study rooms of the department. After reading literature and considering the purpose of the interview, the initial draft of the interview outline was finally determined through expert group meetings. After conducting pre-interviews with 4 nursing students and 2 teachers, the interview outline was finally determined. The two groups of teacher-student interviews had the same outline, both focusing on the teaching/learning process, results, and personal feelings and combined with the satisfaction survey content. The specific interview questions for nursing students are as follows: (1) Learning effectiveness: What has changed your abilities (theoretical, operational, and comprehensive) after teaching ward rounds? (2) Learning process: Are you satisfied with your teaching task allocation? What are your opinions and suggestions? What is the most interesting and difficult part for you? (3) Personal feelings: Do you feel pressure during the learning process? What are the specific manifestations? What advantages do you think this teaching round has, what areas need improvement, and why? The specific interview questions for teachers are as follows: (1) Teaching effectiveness: Can you achieve the goal of training students through teaching rounds? (2) Teaching process: Are you satisfied with the student’s performance during the teaching process? Please share your specific personal thoughts. (3) Personal feelings: Do you think the current teaching rounds can improve the personal abilities of nursing students? What is the most difficult part for you?

### 2.4 Statistical analysis

The quantitative data was analyzed using SPSS 24.0 software. Continuous variables were expressed as the median (25th, 75th percentile) and checked using the Mann–Whitney *U*-test because of the skewed distribution of these variables. Categorical data were characterized as numbers with percentages, and comparisons between groups were performed using the chi-square test. Counting grade data were tested with Wilcoxon rank-sum test. *p <* 0.05 was considered a statistically significant difference.

#### 2.4.1 For the qualitative part

The data recording was promptly transcribed within 24 h following the completion of the interview. Two researchers with certification in qualitative research conducted independent inductive analysis around three themes: teaching effectiveness (self-evaluation of teachers and students), teaching process (classroom performance of students), and personal feelings of teachers and students (pressure). The analysis process involved steps such as promptly transcribing the recording into text, extracting statements relevant to the research topic, encoding repetitive and significant ideas, gathering coding ideas, writing detailed and missing descriptions, identifying similar ideas, elevating thematic concepts, and finally, returning the results to the respondents for verification.

In cases of disagreement, a third researcher participated. Researchers combined qualitative results (interviews) with satisfaction analysis to compare the differences between two teaching methods in personal feelings, self-evaluation, teaching/learning pressure, and other aspects.

## 3 Results

### 3.1 Baseline patient characteristics

The baseline characteristics between the experimental and control groups are presented in Table 3. The experimental group consisted of 115 males and 15 females, with an average age of 23 years, showing no significant difference compared to the control group (all *p* > 0.05). Similarly, no statistical differences were observed between the two groups in terms of educational level and pretest scores (all *p* > 0.05).

**Table 3:** Characteristics of the participants of the two groups.

Characteristics	Control group	Experimental group	*P*
	*N* = 130	*N* = 130	
Sex			0.542
Male, *n* (%)	118 (90.77%)	115 (88.46%)	
Female, *n* (%)	12 (9.23%)	15 (11.54%)	
Age (years)	23.0 (21.0, 23.0)	23.0 (22.0, 23.0)	0.160
Education level			0.228
Junior college graduate	85 (65.38%)	94 (72.31%)	
College graduate	45 (34.62%)	36 (27.69%)	
Pre-admissions test scores			
Theoretical scores	66.5 (64.0, 71.0)	67.0 (64.0, 71.0)	0.842
Operating scores	77.9 (76.2, 80.2)	77.3 (75.7, 79.0)	0.073

### 3.2 Quantitative results

To evaluate the effectiveness of the BOPPPS teaching strategy, the theoretical, practical, and total examination scores of the two groups were compared, as shown in Table 4. The results indicated that the experimental group had significantly higher theoretical, practical, and total examination scores compared to the control group (all *p* < 0.05).

**Table 4:** Comparison of the examination scores between experimental and control groups after training.

	Control group	Experimental group	*P*
	*N* = 130	*N* = 130	
Theoretical scores	75.0 (69.0, 78.8)	77.0 (72.0, 81.0)	<0.001
Operating scores	82.4 (81.4, 84.8)	84.1 (81.6, 87.0)	0.002
Total examination scores	78.7 (75.9, 81.1)	81.1 (78.3, 82.9)	<0.001

Table 5 displays the satisfaction scores of the two groups, including the teachers and students. Students in the experimental group showed higher levels of satisfaction than those in the control group (*p* < 0.001), while no difference in teacher satisfaction scores was observed between the two groups (*p* = 0.323).

**Table 5:** Comparison of satisfaction assessment between experimental and control groups.

	Control group	Experimental group	*P*
	*N* = 130	*N* = 130	
Students’ satisfaction	88.7 (86.7, 89.9)	89.5 (87.5, 92.0)	<0.001
Teachers’ satisfaction	86.5 (84.7, 88.4)	85.5 (83.8, 88.6)	0.323

**Figure 2:**
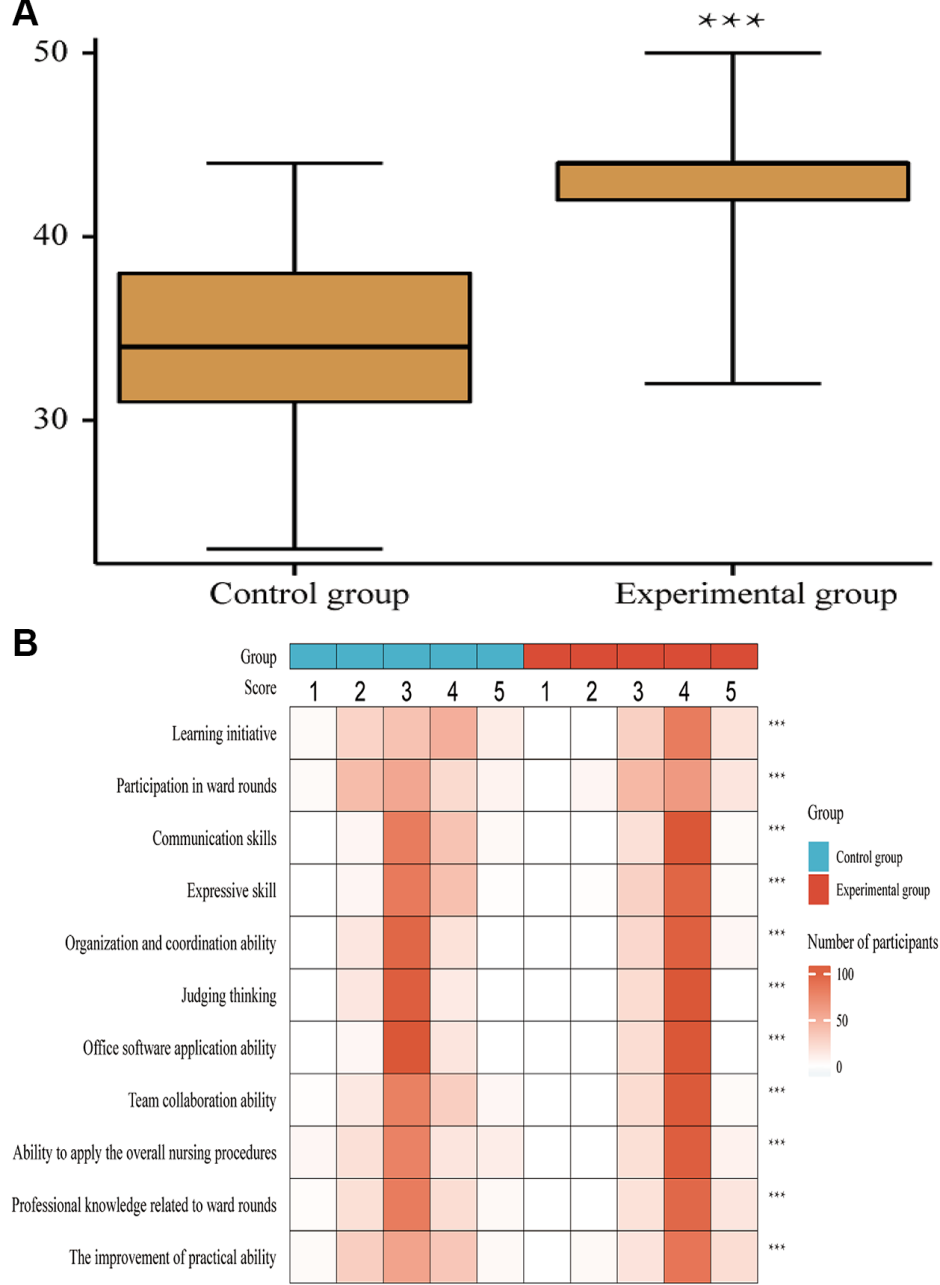
Comprehensive ability scores of two teaching models between the two groups. Panel **(A)** showed the overall comprehensive ability comparison between two groups, with a total score of 11–55 points. Panel **(B)** presented the comprehensive evaluation of 11 aspects between the two group. Likert five-level scoring method was used as the evaluation criterion. A score of 1–5 indicates a lower to higher comprehensive clinical ability. ***p < 0.001.

With the advances in education quality, evaluating the students’ competency represents an important method of assessing teaching efficacy. The comprehensive ability of the students was evaluated throughout the whole teaching process. The results showed that students in the experimental group scored higher than those in the control group (*p* < 0.001, Figure 2A). The evaluation of the students’ comprehensive ability was conducted on 11 aspects, including learning initiative, participation in ward rounds, communication skills, skill of expression, organization and coordination ability, critical thinking, office software application ability, team collaboration, application of the overall nursing procedures, professional knowledge related to ward rounds, and the improvement of practical ability (all *p* < 0.001, Figure 2B).

### 3.3 Qualitative results

#### 3.3.1 Effect evaluation

Both the experimental group and the control group of nursing students believe that teaching ward rounds has improved their abilities. The experimental group of nursing students provided feedback that their professional and comprehensive abilities have been improved. The control group lacked feedback on comprehensive skills.

“I have learned auscultation of breath sounds” (N4, 26); “I have learned about the nursing and observation of closed thoracic drainage tubes” (N77, 90); “I have learned a lot of things beyond textbooks through teaching ward rounds, such as PowerPoint production methods, literature review, and medical history reporting” (N10); “I found that there were many issues with my previous nursing records” (N147) (Table 6).

**Table 6:** Interview.

Groups	Roles	Items	Quotations
Control group	Teachers	Teaching effectiveness	(1) “Students have gained a deeper understanding of the key points of postoperative observation and nursing for lung cancer;” (2) “Students do not know how to establish a nurse–patient relationship;” (3) “Medical history reporting lacks logical coherence and highlights key points;” (4) “The students in this group were unwilling to take the initiative.”
		Teaching process	(1) “The discussion topic is not proactive enough. Only the team leader and responsible nursing students participate in the discussion;” (2) “Other classmates in the group had poor grasp of basic knowledge and were not actively engaged in discussions.”
		Personal feelings	(1) “Many students have forgotten about the knowledge and need teachers to explain it in class;” (2) “There were significant differences among students, and students with poor foundations find it difficult to participate in discussions.”
	Nursing students	Teaching effectiveness	(1) “Understand the methods of auscultating respiratory sounds in the lungs, but independent operation still requires the assistance of a teacher;” (2) “Learned how to read chest X-rays.”
		Teaching process	“I feel like the teacher talked a lot about things that are similar to the classroom content.”
		Personal feelings	“I hope the teacher can systematically review the basic knowledge before teaching ward rounds, as many of the knowledge of lung anatomy had been forgotten.”
Experimental group	Teachers	Teaching effectiveness	(1) “The medical history report was relatively complete, able to highlight key points, and the PPT production was relatively standard, indicating that nursing students actively think and analyze during the production process;” (2) “The ability to distinguish between dry and wet rales, and the ability to auscultate lung breathing has significantly improved;” (3) “The team leader coordinated their work very well.”
		Teaching process	(1) “Able to communicate effectively with patients and their families during ward rounds.” (2) Active group discussion
		Personal feelings	(1) “The students in this group demonstrated excellent communication skills, literature review, and expression abilities;” (2) “Students actively think;” (3) “There is too much preparation work for teaching, and I often feel tired and had insufficient time;” (4) “Setting up a teacher’s specialized position and separating from clinical work during teaching;”
	Nursing students	Teaching effectiveness	(1) “I have learned a lot beyond textbooks, such as making PowerPoint presentations, reviewing literature, and reporting medical history;” (2) “I have learned how to auscultate respiratory sounds, but I still need to accumulate more clinical experience;” (3) “I am aware of the nursing and observation of chest drains;” (4) “I found that there were many issues with the nursing records I wrote before.”
		Teaching process	(1) “Everyone speaks positively;” (2) “The teacher recognized my different opinions and felt a great sense of achievement;” (3) “During the initial preparation, I received a lot of advice from experienced nurses and doctors.”
		Personal feelings	(1) “Through literature review, I have gained many new ideas;” (2) “Can you provide some free literature search platforms? Students are under great financial pressure to pay for them;” (3) “I need to spend a lot of personal time studying while working in clinical settings, and I feel a lot of pressure;” (4) “Can you provide a few days for learning;”

#### 3.3.2 Student performance

Both groups of teachers and students provided feedback indicating that the experimental group of nursing students was more active in classroom speeches and discussions, and the experimental group of nurses engaged in active learning and independent thinking during this process.

“The teacher recognized my different opinions and felt a great sense of achievement” (N132); “A classmate proposed a different idea, and I think it makes sense” (N306); “In the early stages of preparation, I received a lot of important advice from experienced nurses and doctors” (N247).

The control group of nursing students showed relatively indifference during the interview and did not respond actively enough to the interview questions. From the feedback, it can be inferred that some students believe that teaching rounds are relatively difficult.

“I feel that case reporting is a bit difficult” (N206); “I do not know how to communicate to obtain the patient’s consent and cooperation,” (N171); “Only the team leader and responsible nursing students participate in the discussion” (N303); “No classmates took the initiative to ask questions” (N301).

#### 3.3.3 Personal feelings

The experimental group teachers need to complete a lot of preparation and counseling work in the early stage of ward rounds, which results in their energy and time exceeding the normal work level, leading to a sharp increase in work pressure.

“There is too much preparation work for teaching, and I often reply to students’ questions after returning home, feeling that my energy and time are not enough” (N310); “Some students have poor foundations, which led to heavy tutoring tasks for me in the early stage” (N312); I suggest setting up a teacher specialized position and separating from clinical work during teaching (N308); I suggest adding a teaching secretary (N312).

Students need to invest a significant amount of personal time and effort, and even economic costs, to acquire knowledge and skills. The feedback from nursing students in the experimental group was more significant.

“Can you provide me with some free search platforms? Obtaining literature often requires payment, which increases my financial pressure” (N08); “Besides the pressure of clinical work, I have to spend a lot of personal time studying, which makes me feel very tired” (N32); Can you provide me with a few more days to study? (N70).

## 4 Discussion

This research is the first to apply the BOPPPS model to standardized training of nurses and indicated that the BOPPPS model has obvious advantages over traditional teaching methods in developing theoretical knowledge, practical skills, and comprehensive ability.

Nursing students often lack a comprehensive understanding of holistic nursing when entering clinical practice, hindering the application of theoretical knowledge to clinical practice. Teaching ward rounds are an important teaching method used to connect theoretical knowledge and clinical ability. The teaching ward rounds deepen theoretical knowledge and cultivate clinical thinking. In contrast, traditional teaching methods mostly consist of simple teacher-led teaching. The BOPPPS model is a student-centered teaching method and observation system, aiming to cultivate professional and personal ability; furthermore, it focuses on the effectiveness and diversity of teaching. The BOPPPS model has been repeatedly reported in clinical medicine teaching (17). For the first time, the BOPPPS model was applied in standardized nursing training, proving that the system can improve the overall ability of nursing students, including theoretical knowledge, practical skills, and comprehensive ability. Compared with traditional learning, the BOPPPS model requires students to review before the class, and teachers to complete the pre-class evaluation and guidance, which may minimize the knowledge disparity between students. In addition, throughout the entire teaching process, teachers can continuously receive feedback from students, adjust the teaching rhythm in a timely manner, and improve teaching methods (17). Prior studies have established the efficacy of the BOPPPS teaching model in diverse educational settings (7, 13–15). Nonetheless, there is a gap in the literature regarding its implementation in standardized nurse training programs. Our research employs qualitative and quantitative methodologies to showcase the superior benefits of the BOPPPS model compared to conventional teaching approaches in enhancing theoretical knowledge, practical skills, and the comprehensive ability of newly recruited nurses.

Combined with the Bruner theory, the BOPPPS model constantly stimulates students’ motivation for learning. Bruner reported that intrinsic motivation is the real motivation for learning, which can be divided into four categories, including curiosity, competence, complacency, and reciprocity (18). In the BOPPPS model, the first step is to stimulate students’ curiosity by introducing cases. Moreover, pre-class assessment and targeted guidance can improve the personal ability of nursing students. Finally, teamwork can help teachers guide students to actively participate in learning through the inner motivation of interacting with people, which conforms to reciprocity. Meanwhile, introducing nursing students to “lecturer” roles stimulates the learning initiative of nursing students. This makes students more involved in the learning process and is beneficial for improving teaching effectiveness.

In the whole teaching process, students are enabled to show their professional knowledge, skills, and comprehensive ability, including communication. With the development of medical technology, the quality of medical services is gradually declining. Nurses spend less time communicating with patients, and the lack of humanistic care violates the essence of nursing care. Although many studies have shown that medical education must focus on cultivating and evaluating soft skills, the current nursing education system does not support such processes. Studies have shown that the personal comprehensive ability of nurses is directly related to improving nursing quality (19, 20). Therefore, strengthening the social and personal skills of nursing students has become a priority (21, 22). Currently, standardized training is still limited to the cultivation of theoretical knowledge and practical skills, and there is a lack of soft skills training for nursing students. This study revealed that the comprehensive ability of students in the BOPPPS model group was higher than those in the traditional teaching group, which is consistent with Hu et al.’s (23) study. Specifically, the nursing students in the experimental group performed better in communication, collaboration, critical thinking, and other aspects. The BOPPPS model not only stresses the application of clinical professional knowledge and skills but also emphasizes the cultivation of individual non-technical abilities. Nursing students were required to show proficiency in office software operation, review literature, and help complete team tasks. In this process, nursing students need to coordinate, organize, plan, communicate, and do other activities, which cultivates their comprehensive abilities. The BOPPPS model is aligned with the requirements of standardized training for nurses in improving the quality of nursing training. The study results are consistent with Ma et al.’s (17) study.

The lack of teaching resources has always been a problem in medical teaching. Medical staff devoted less and less time to clinical teaching (24), which is associated with increased demand and busy clinical work. The interview showed that teachers and nursing students were more inclined to the BOPPPS model, indicating that it had better student participation and teaching feedback, and had more advantages in terms of personal ability cultivation. However, we found no difference in satisfaction scores. Analysis of the reasons can be found that under the existing clinical training management mode, adopting this model increased the work and study pressure for teachers and nursing students. Clinical teaching is usually completed by clinical nurses part-time, but in the BOPPPS model design, teachers needed to do a lot of teaching work in the whole cycle stage of ward rounds, including the early evaluation and guidance, guidance and supplement in the process, as well as later evaluation and summary, which need full-time staff to complete. Therefore, teachers have to occupy their private time to ensure the quality of teaching. The same problem also exists for nursing students. Ward round is always interspersed with clinical work, and nursing students had to complete their spare time. The double pressure of work pressure and study pressure of new nurses affects students’ satisfaction. Therefore, the impact of human resources and the arrangement of teaching time on satisfaction needs further research.

Nevertheless, the limitations of the study should be acknowledged. First, the sample size was small, with only 260 students enrolled in the study. Second, there is no standard guide for the application of the BOPPPS model in medical disciplines, and there is no standard for the effective evaluation of the BOPPPS model in China. Therefore, whether the teaching process design meets the standard, and the scientific evaluation methods need to be further discussed. Third, there is a lack of discussion on the factors affecting teaching efficacy, which require further analysis and research. Finally, the ultimate goal of cultivating nursing students is to improve patient safety and enhance their medical experience. However, there is a lack of investigation at the patient level in our study, and further research is needed to demonstrate the effectiveness of this model from multiple aspects.

## 5 Conclusion

In conclusion, the BOPPPS model is more effective than traditional teaching methods in developing nursing students’ practical skills, theoretical knowledge, and comprehensive ability. This model may improve standardized training for nurses in China. However, further high-quality studies are needed to evaluate the effectiveness of the BOPPPS model in standardized training for nurses.

## Data availability statement

The datasets presented in this article are not readily available due to data restrictions by the authors. Requests to access the datasets should be directed to SY, enqo1982@outlook.com.

## Ethics statement

The studies involving humans were approved by The Ethics Committee of the First Affiliated Hospital of Chongqing Medical University. The studies were conducted in accordance with the local legislation and institutional requirements. Written informed consent for participation was not required from the participants or the participants’ legal guardians/next of kin in accordance with the national legislation and institutional requirements not to be credited with authorship.

## Author contributions

YS: Writing – review & editing, Writing – original draft, Supervision, Software, Resources, Project administration, Methodology, Investigation, Formal analysis, Data curation, Conceptualization.

## References

[1] General Office of China National Health and Family Planning Commission. (2016). Notice of the General Office of the National Health and Family Planning Commission of China on Issuing the “Training Program for New Nurses (Trial)”. Available at: http://www.nhc.gov.cn/yzygj/s3593/201602/91b5a8fa3c9a45859b036558a5073875.shtml?from=groupmessage&isappinstalled=1

[2] Jiang X Liu D. Clinical nurses’ standardization training status and needs. Chin Nurs Managment. (2012) 12:50–2.

[3] Jiao J Xu K Ying Q Li Z Wang W Wu Y. Construction of the evaluation system for the effects of standardized training of new nurses. Chin J Nurs. (2019) 54:1285–90. doi: 10.3761/j.issn.0254-1769.2019.09.001

[4] Falk K Falk H Jakobsson UE. When practice precedes theory – a mixed methods evaluation of students’ learning experiences in an undergraduate study program in nursing. Nurse Educ Pract. (2016) 16:14–9. doi: 10.1016/j.nepr.2015.05.010, PMID: 26070493

[5] Lam TP Wan XH Ip MS. Current perspectives on medical education in China. Med Educ. (2006) 40:940–9. doi: 10.1111/j.1365-2929.2006.02552.x, PMID: 16987183

[6] Bi M Zhao Z Yang J Wang Y. Comparison of case-based learning and traditional method in teaching postgraduate students of medical oncology. Med Teach. (2019) 41:1124–8. doi: 10.1080/0142159X.2019.1617414, PMID: 31215320

[7] Chen L Tang XJ Chen XK Ke N Liu Q. Effect of the BOPPPS model combined with case-based learning versus lecture-based learning on ophthalmology education for five-year paediatric undergraduates in Southwest China. BMC Med Educ. (2022) 22:437. doi: 10.1186/s12909-022-03514-4 35668389 PMC9170341

[8] Hsieh MC Chen TY. Promoting innovation in the objective structured teaching examination and feedback: clustering teachers to aid teaching evaluation. Med Educ Online. (2019) 24:1620544. doi: 10.1080/10872981.2019.1620544, PMID: 31184288 PMC6567259

[9] Pattison P Russell D. Instructional skills workshop handbook. Vancouver, Canada: UBC Centre for Teaching and Academic Growth (2006).

[10] Yang Y You J Wu J Hu C Shao L. The effect of microteaching combined with the BOPPPS model on dental materials education for Predoctoral dental students. J Dent Educ. (2019) 83:567–74. doi: 10.21815/JDE.019.068, PMID: 30858273

[11] Li Z Cai X Zhou K Qin J Zhang J Yang Q. Effects of BOPPPS combined with TBL in surgical nursing for nursing undergraduates: a mixed-method study. BMC Nurs. (2023) 22:133. doi: 10.1186/s12912-023-01281-1, PMID: 37088853 PMC10122814

[12] Xu Z Che X Yang X Wang X. Application of the hybrid BOPPPS teaching model in clinical internships in gynecology. BMC Med Educ. (2023) 23:465. doi: 10.1186/s12909-023-04455-2, PMID: 37349730 PMC10286474

[13] Liu XY Lu C Zhu H Wang X Jia S Zhang Y. Assessment of the effectiveness of BOPPPS-based hybrid teaching model in physiology education. BMC Med Educ. (2022) 22:217. doi: 10.1186/s12909-022-03269-y, PMID: 35354465 PMC8966603

[14] Wen H Xu W Chen F Jiang X Zhang R Zeng J. Application of the BOPPPS-CBL model in electrocardiogram teaching for nursing students: a randomized comparison. BMC Med Educ. (2023) 23:987. doi: 10.1186/s12909-023-04983-x, PMID: 38129836 PMC10740289

[15] Ma X Ma X Li L Luo X Zhang H Liu Y. Effect of blended learning with BOPPPS model on Chinese student outcomes and perceptions in an introduction course of health services management. Adv Physiol Educ. (2021) 45:409–17. doi: 10.1152/advan.00180.2020, PMID: 34018832

[16] Chongqing Municipal Health and Family Planning Commission. (2014). Notice on developing standardized training for nurses. Available at: www.cq.gov.cn/ywdt/zwhd/bmdt/202205/t20220520_10737925.html

[17] Ma X Zeng D Wang J Xu K Li L. Effectiveness of bridge-in, objective, pre-assessment, participatory learning, post-assessment, and summary teaching strategy in Chinese medical education: a systematic review and meta-analysis. Front Med. (2022) 9:975229. doi: 10.3389/fmed.2022.975229, PMID: 36186766 PMC9521335

[18] Bruner JS. The process of education Harvard University Press (1960). Chinese Nursing Journal.

[19] Wenhai F Zheng D Xinwei F Xiuya L. Clinical nursing teachers’ soft skills and their effects on teaching behaviors. Chin J Nurs. (2019) 54:1070–4. doi: 10.3761/j.issn.0254-1769.2019.07.022

[20] Kaiafas KN. Emotional intelligence and role-modeling Nursing's soft skills. J Christ Nurs. (2021) 38:240–3. doi: 10.1097/CNJ.0000000000000881, PMID: 34477586

[21] Liebrecht C Montenery S. Use of simulated psychosocial role-playing to enhance nursing Students' development of soft skills. Creat Nurs. (2016) 22:171–5. doi: 10.1891/1078-4535.22.3.171, PMID: 29195526

[22] Parreira P Santos-Costa P Graveto J Ferreira PA Salgueiro-Oliveira A Sousa LB. Personal and technological skills to coach people with non-communicable diseases: development and validation of a scale for nursing students. Heliyon. (2021) 7:e06140. doi: 10.1016/j.heliyon.2021.e06140, PMID: 33644450 PMC7889991

[23] Hu K Ma RJ Ma C Zheng QK Sun ZG. Comparison of the BOPPPS model and traditional instructional approaches in thoracic surgery education. BMC Med Educ. (2022) 22:447. doi: 10.1186/s12909-022-03526-0, PMID: 35681190 PMC9185859

[24] Bell PR Hampton S. No time for teaching at our teaching hospitals. Ulster Med J. (2014) 83:118–9. 25075143 PMC4113158

